# Recovery of Polyphenols from Brewer’s Spent Grains

**DOI:** 10.3390/antiox8090380

**Published:** 2019-09-07

**Authors:** Rares I. Birsan, Peter Wilde, Keith W. Waldron, Dilip K. Rai

**Affiliations:** 1 Department of Food BioSciences, Teagasc Food Research Centre Ashtown, D15KN3K Dublin, Ireland; 2 Food Innovation and Health Programme, Quadram Institute Bioscience, Norwich Research Park, Colney NR4 7UQ, UK; 3 Anglia Science Writing Ltd., Wramplingham NR18 0RU, Norfolk, UK

**Keywords:** brewer’s spent grain, polyphenols, microwave assisted extraction, ultrasound assisted extraction, liquid chromatography-mass spectrometry

## Abstract

The recovery of antioxidant polyphenols from light, dark and mix brewer’s spent grain (BSG) using conventional maceration, microwave and ultrasound assisted extraction was investigated. Total polyphenols were measured in the crude (60% acetone), liquor extracts (saponified with 0.75% NaOH) and in their acidified ethyl acetate (EtOAc) partitioned fractions both by spectrophotometry involving Folin–Ciocalteu reagent and liquid-chromatography-tandem mass spectrometry (LC-MS/MS) methods. Irrespective of the extraction methods used, saponification of BSG yielded higher polyphenols than in the crude extracts. The EtOAc fractionations yielded the highest total phenolic content (TPC) ranging from 3.01 ± 0.19 to 4.71 ± 0.28 mg gallic acid equivalent per g of BSG dry weight. The corresponding total polyphenols quantified by LC-MS/MS ranged from 549.9 ± 41.5 to 2741.1 ± 5.2 µg/g of BSG dry weight. Microwave and ultrasound with the parameters and equipment used did not improve the total polyphenol yield when compared to the conventional maceration method. Furthermore, the spectrophotometric quantification of the liquors overestimated the TPC, while the LC-MS/MS quantification gave a closer representation of the total polyphenols in all the extracts. The total polyphenols were in the following order in the EtOAc fractions: BSG light > BSG Mix > BSG dark, and thus suggested BSG light as a sustainable, low cost source of natural antioxidants that may be tapped for applications in food and phytopharmaceutical industries.

## 1. Introduction

Brewers’ spent grain (BSG) is generated in millions of tonnes every year as the major by-product of the brewing industry, with an annual global production estimated to be 39 million tonnes, of which EU generates ~8 million tonnes [1,2]. BSG is used as a low-value animal feed with a market value of ~35 Euro/tonne and thus making it an ideal substrate from which to recover high value compounds [3]. In addition to cellulose, hemicellulose, lignin, protein and lipids as the main components, BSG also contains low molecular weight phenolic compounds that have been associated with a wide array of health-benefiting properties [4,5].

A number of extraction methods, optimized and applied towards the recovery of polyphenols from BSG, have been comprehensively reviewed by several authors [3,6,7]. Depending on the types of BSG produced as a result of different cooking temperatures (70–250 °C), the polyphenol contents also differ between the lightly roasted malt producing light or pale BSG and the deeply roasted malts producing dark or black BSG. A common practice in breweries is to mix the light and dark malts in the ratio ~9:1 *w*/*w* in order to obtain the desired caramel colour and aroma of the beverage. Since BSG predominantly contains bound phenolics, chemical or enzymatic hydrolysis protocols are routinely used to release the phytochemicals bound to the cellular-wall components [8,9,10]. Solvent extraction or chemical hydrolysis combined with ultrasound (UAE) or microwave assisted extraction (MAE) or other physical cell-disruption techniques have been shown to increase the extraction yield of targeted compounds from BSG and similar biomass [7,11,12,13]. For example, in the recovery of BSG polyphenols, an optimised MAE method has been reported to result in a five-fold higher ferulic acid yield than the conventional solid–liquid extraction techniques [14]. In contrast, the same MAE parameters were also applied by Stefanello et al. [15] on BSG and corn silage, but the MAE yielded significantly lower total phenolic content than the conventional maceration method. In a separate study, mathematical models were used to optimize three extraction parameters (i.e., substrate to solvent ratio, extraction temperature and solvent composition) for MAE and UAE to recover maximum yield of unbound polyphenols from the unsaponified BSG. The subsequent experiments performed using the optimum parameters also resulted in higher polyphenolic contents by UAE (4.1 mg GAE/g BSG dw) and by MAE (3.9 mg GAE/g BSG dw) compared with the maceration method (3.6 mg GAE/g BSG dw) [15]. Both MAE, based on rapid heating of the solvent through microwave energy (that causes molecular motion via ionic conduction and dipole rotation), and UAE based on acoustic cavitation, increase the solvent penetration into the substrate leading to improved mass transfer rates. There is, however, a limited number of studies that focus on the UAE, MAE and conventional extraction methods to recover polyphenols from saponified BSG despite the presence of optimisation studies on individual methods in BSG [14,15,16] or similar substrates [17,18]. In addition, several of the aforementioned and other BSG polyphenol extraction studies were quantified spectrophotometrically using the Folin–Ciocalteu (FC) chemical method [15,16,19,20,21,22] either alone or with hyphenated chromatographic methods [15,16,20,22,23,24,25,26]. The spectrophotometric methods suffer generally by over estimating the phenolic contents since other non-polyphenolic molecules (e.g., reducing sugars) interact with the FC reagent used in the assay [27,28]. It is for this reason that in recent years researchers are discouraged from quantifying polyphenols using only the spectrophotometric methods [29,30].

In this study, we have investigated and compared the recovery of polyphenols from saponified light (L), dark (D) and BSG Mix using maceration, MAE and UAE techniques. The parameters for the various extraction methods have been adapted from the literature for maceration and UAE, whereas previously optimised parameters were applied for MAE. The objective of this study is to assess the polyphenol recovery from each type of BSG substrate using three different extraction methods. In addition, we have evaluated the enrichment of polyphenols through liquid-liquid partitioning of acidified ‘liquors’ (saponified fractions), which has been reported to lesser degree. Both the spectrophotometric and the LC-MS/MS methods have been employed and compared for the quantification of polyphenols in the various BSG fractions.

## 2. Materials and Methods

### 2.1. Materials and Chemicals

BSG L and D were provided by Diageo Ireland, Dublin. BSG Mix (light:dark, ~9:1 *w*/*w*) was obtained from the River Rye Brewing Company, Cellbridge, County Kildare, Ireland. The BSG samples were directly transported to the research centre within 30 min., oven-dried (Binder E28 oven, 72 h, 60 °C), milled (<1 mm) and vacuum packed until required.

The organic solvents (methanol, acetone, ethyl acetate (EtOAc), formic acid, acetonitrile), and sodium hydroxide (NaOH) were purchased from Merck (formerly Sigma Aldrich, Arklow, Co. Wicklow, Ireland). Polyphenol standards of gallic acid, *p*-coumaric acid, ferulic acid, sinapic acid, caffeic acid, protocatechuic acid, 4-hydroxybenzoic acid and +(-)catechin; and the chemicals FC reagent, hydrochloric acid and sodium carbonate were purchased from Merck (Arklow, Co. Wicklow, Ireland). Leucine-enkelphine was purchased from VWR International Ltd. (Blanchardstown, Dublin, Ireland).

### 2.2. Solid-Liquid Extraction

**Figure 1:**
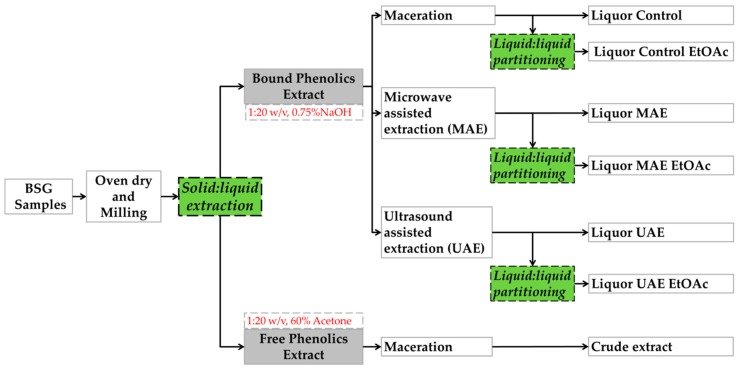
Flow chart showing the extraction procedure for brewers’ spent grain (BSG) samples (light (L), dark (D), and Mix) for free phenolics and bound phenolics. Alkali-hydrolysed fractions (liquors) were partitioned with ethyl acetate (EtOAc).

A schematic flow of the extraction procedure used is illustrated in Figure 1. Extraction of free (unbound) polyphenols referred to as crude extracts from BSG samples was carried out as in the previously optimised method [16], where 3 g milled BSG was mixed with 60 mL of 60% acetone at 60 °C for 30 min. with constant stirring. For the extraction of bound phenolics, 0.75% NaOH aqueous solution at 80 °C for 30 min. with constant stirring was used [14,18].

### 2.3. Microwave Assisted Extraction

Microwave assisted extractions of BSG phenolics were performed according to the method previously optimized and reported by Moreira et al. [14]. The extraction was carried out in a microwave MARS^TM^-6 (CEM, Matthews, NC, USA) equipped with a 40-position carousel. 2 g BSG samples were transferred to TFM extraction vessels with 40 mL alkali solution. Extraction was carried out for a duration of 15 min. at 100 °C. In all the vessels magnetic stirrers were added and used at maximum stirring speeds, while the pressure-leak and temperature were monitored for each vessel.

### 2.4. Ultrasound Assisted Extraction

Ultrasound assisted extraction was carried out on the Transonic TI-H-10 35 kHz sonication bath (ELMA Sch., Singen, Germany) at ~80 °C for 30 min. adapting the parameters previously optimised [17,18] in similar substrates. The substrate to solvent ratio (1:20 *w*/*v*) and the alkali concentration were maintained as used in the MAE and maceration methods, where 2.5 g BSG samples were mixed with 50 mL 0.75% NaOH solution in 100 mL amber bottles. The bottles were sealed to avoid any loss of solvents.

### 2.5. Preparation of Samples Following Maceration, MAE and UAE Treatments

After the extraction times were complete, all the extracts were left to cool at room temperature followed by centrifugation at 8400 rpm for 10 min. (MegaStar 600, VWR, Leuven, Belgium). The supernatants were pooled and syringe filtered through 0.45 μm PTFE filters for free phenolics, and PVDF filters for bound phenolic extracts. Aliquots (20 mL) of the liquor supernatants were acidified by adding hydrochloric acid solution (37%) until the pH reached 6.5 and subsequently subjected to liquid-liquid partitioning in EtOAc:water (1:1 *v*/*v*, 3 times) to obtain polyphenol-enriched fractions. The EtOAc fractions were evaporated to dryness under nitrogen and reconstituted in 20 mL 50% methanol. All the extractions were carried out in triplicate and stored at −25 °C until further use.

### 2.6. Total Phenolic Content Assay

Total phenolic content of BSG extracts was determined by colorimetric assay using FC reagent following [31]. Briefly, in 1.5 mL Eppendorf tube, 100 µL of extract was mixed with 100 µL each of methanol and FC reagent, and 700 µL of 20% sodium carbonate solution. The tubes were vortexed and incubated for 20 min. in darkness at room temperature. After the incubation, the tubes were centrifuged at 13,000 rpm for 3 min. to remove turbidity. Following this, 200 µL of the reaction mixture was transferred into 96-well micro plate and measured for absorbance at 735 nm using a spectrophotometer (FLUOstar Omega, BMG Labtech, Germany). Different concentrations of gallic acid as standards were used (10–300 µg/mL in 50% methanol) to prepare a calibration curve. The results are expressed in milligrams of gallic acid equivalent per gram dry weight (mg GAE/g BSG dw) BSG.

### 2.7. LC-MS/MS Identification and Quantification of BSG Phenolic Compounds

Quadrupole time-of-flight (Q-ToF) Premier mass spectrometer coupled to Alliance 2695 HPLC system (Waters Corporation, Milford, MA, USA) was used to profile various phytochemicals in the BSG L EtOAc fraction following the procedure previously described [32]. Accurate mass measurements of the molecular ions were achieved using an internal reference compound (Leucine–Enkephalin). The separation of the compounds was achieved on an Atlantis T3 C18 column (100 × 2.1 mm; 3 µm) using milliQ water (solvent A) and acetonitrile (solvent B) both containing 0.1% formic acid at a flow rate of 0.3 mL/min. at 40 °C. Electrospray ionisation (ESI) mass spectra were recorded on a negative ion mode for a mass range *m*/*z* 70–1000. Capillary and cone voltages were set at 3 kV and 30 V, respectively. Collision-induced dissociation (CID) of the analytes was performed using argon at 12–20 eV. Ultra-high performance liquid chromatography coupled to tandem quadrupole mass spectrometer (UPLC-TQD, Waters Corp., Milford, MA, USA) was used to quantify the BSG polyphenols by adapting the previous method used in raw barley [33]. Separation of the phenolics was carried out on an Acquity UPLC HSS T3 column (2.1 × 100 mm, 1.8 µm). The mobile phase consisted of milliQ water (solvent A) and acetonitrile (solvent B) both containing 0.1% formic acid. The UPLC separation was performed by an increasing organic solvent gradient from 2% to 98% B at a flow rate of 0.5 mL/min. for 10 min. The column temperature was set at 50 °C, while the samples were kept at 4 °C. The ESI source was set in negative mode and the quantification of each compound was performed using multiple reaction monitoring (MRM) method (Supplementary Table S1). 

For the quantification of polyphenols, a stock solution (1000 ppm) for each standard was prepared and appropriate dilutions covering the range of 0.098 to 100 ppm were made to obtain standard curves. Targetlynx^TM^ integration software (Waters Corp., Milford, CT, USA) was used to quantify the compounds in the various extracts.

### 2.8. Statistical Analysis

Results are expressed as means of the triplicates ± standard deviation (SD). Differences between means were analysed using one-way analysis of variance with post-hoc Tukey test (SPSS Statistics 24). The statistical analysis on the different groupings was carried out using Minitab 18.0 (Minitab, Inc., State College, Pennsylvania, USA). The values were considered significantly different when *p* < 0.05.

## 3. Results and Discussion

### 3.1. Total Phenolic Content

The total phenolic content (TPC) from the crude extracts for the L, D and Mix BSG were 2.84 ± 0.11, 2.81 ± 0.14 and 3.85 ± 0.04 mg GAE/g BSG dw, respectively (Table 1). Past studies, by other authors, on the crude extracts of light and dark BSG have also shown TPC in a similar range [24,34]. These relatively low TPC levels in the crude extracts are because of the fact that the BSG contains a high amount of lignin ranging from 19.4–49.2 g/100 g that is connected to its cell wall polysaccharides by phenolic acids [10,16,35]. Therefore, it is essential to hydrolyse the rigid lignocellulose structural components to release the phenolic acids. Alkali hydrolysis is commonly used with BSG and other similar substrates. The TPC of the hydrolysed fraction (liquor) prior to acidification and partitioning is often reported, which is four- to five- times higher than the TPC values of the crude extracts [24,26]. For example, McCarthy et al. [25] recorded 16.0 mg GAE/g BSG dw and 18.3 mg GAE/g BSG dw for the light and dark BSG liquors, respectively. This trend is also evident from our study, where TPC values for the liquors ranged from 15.42 to 19.20 mg GAE/g BSG dw as opposed to the crude extracts (2.81 to 3.85 mg GAE/g BSG dw). Generally the dark BSG have shown higher levels of TPC values than the light BSG owing to the presence of high molecular weight melanoidins [20], which are accumulated as by-products of the Maillard reaction. The melanoidins mostly consist of sugar degradation products and amino acids [36] that can also react with FC reagent and thus give false elevated TPC.

However, after the acidification of the liquors and subsequent partitioning with EtOAc, the TPC values of the EtOAc ranged between the crude and the liquor fractions (Table 1). Interestingly, the TPC of EtOAc fractions in the BSG D averaging 3.17 mg GAE/g dw is significantly lower than those of the L and Mix BSG averaging 4.23 and 4.52 mg GAE/g dw, respectively. Similar findings where the phenolics were lower in the hydrolysed dark BSG compared to light BSG have been reported by Moreira et al. [26]. Although the application of MAE and UAE techniques resulted, in general, lower TPC in the BSG EtOAc fractions than the conventional maceration method, but this decrease was not statistically significant except between the MAE and control BSG L. The possible reason for this decrease is due to the structural characteristic of the BSG as it predominantly contains a high lignin content [4,10]. It has been suggested before that the MAE is not able to promote sufficient molecular movement and rotation to overcome the lignin-barrier in contrast to constant stirring in the maceration method [14,15]. Furthermore the high temperature in MAE may induce the degradation of thermolabile polyphenols. A study on the effect of temperature on the extraction of polyphenols from *Gordonia axillaris*, an edible wild fruit, has shown a decrease in antioxidants’ recovery with higher temperatures in MAE [37]. In general, high temperature has a positive effect on the extraction yield due to enhanced solubility and diffusivity of materials, however in UAE the high temperature has a negative effect on the extraction yield [38]. The high temperature increases the solvent vapour pressure and results in a decrease in surface tension that affect the cavitation bubble formation, which may explain the low TPC in the UAE treated samples.

### 3.2. LC-MS/MS Identification of BSG Polyphenols

**Figure 2:**
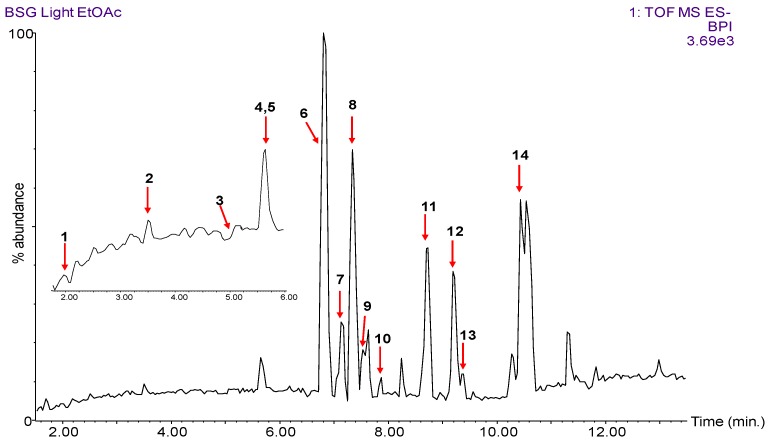
HPLC-Q-ToF (quadrupole time-of-flight) chromatogram of EtOAc fraction of BSG L showing the polyphenols (peaks 1–14) as assigned in Table 2. Shown in the inset is a close-up figure for the minor peaks 1–5. The elution time for peaks 1, 3 and 4 are demonstrated in their extracted ion chromatograms in Supplementary Figure S1.

As many as 14 different polyphenols were tentatively identified in the BSG L EtOAc extract using the accurate mass measurements, fragment ions and in conjunction with the literature (Figure 2, Table 2). Few of these polyphenols (protocatechuic acid and caffeic acid) were present in low amounts or co-eluted (syringic acid) with other phenolic acids as illustrated in the magnified inset in Figure 2 and the extracted ion chromatograms for these compounds in Supplementary Figure S1. Seven phenolic acids (ferulic acid, protocatechuic acid, 4-hydroxybenzoic acid, caffeic acid, syringic acid, p-coumaric acid and sinapic acid) and a flavonoid (catechin) were identified using commercially available standards and subsequently quantified using UPLC-TQD (Section 3.3). Several of the ferulic acid dimers and trimers listed in Table 2 have been identified previously in BSG using HPLC-DAD-MS/MS methods [4,14,39].

**Figure 3:**
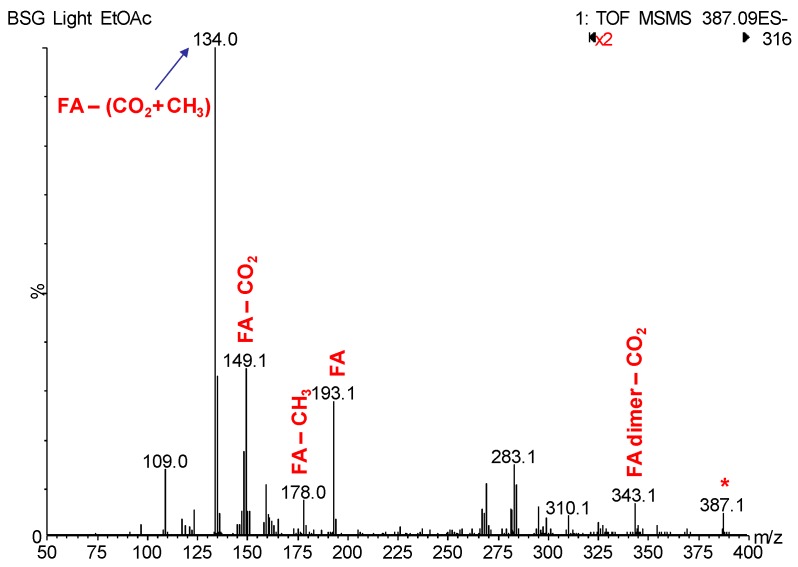
Electrospray ionisation (ESI)-MS/MS of *m*/*z* 387.1 showing the fingerprint fragment ions of the ferulic acid dimer. (FA = ferulic acid).

In this study, an additional peak eluting at 7.13 min (peak 7) showed to contain a cluster of two molecules of ferulic acids corresponding to *m*/*z* 387.1073 [predicted molecular formula (C_20_H_20_O_8_)]. On subjecting this molecular ion to MS/MS, the fragment ions *m*/*z* 343.1 (loss of CO_2_), ferulic acid at *m*/*z* 193.1, *m*/*z* 149.1 (ferulic acid- CO_2_) and *m*/*z* 134.0 (ferulic acid –(CO_2_ CH_3_)) further supported the detection of dimeric ferulic acid (Figure 3). Such non-covalent dimers generally form when the monomeric units are abundant in the sample, i.e., ferulic acid in this case.

### 3.3. UPLC-MS/MS Quantification of BSG Polyphenols

Total polyphenols, the aggregate sum of individual polyphenols measured by UPLC-MS/MS, in each of the BSG EtOAc fractions, were found in decreasing levels of abundance in the following order: BSG L > BSG Mix > BSG D (Table 3). Statistically significant differences were found (in the same direction of abundance as TPC by FC) between the total polyphenols of BSG L, D and the Mix. The BSG L (2,741 µg/g dw) contained more than four times the total polyphenols found in BSG D (693 µg/g dw), which is in contrast to the TPC values where the dark BSG contained similar levels to light BSG as in this study (Table 1) or exceeded the light BSG [20,25]. The BSG Mix showed intermediate total polyphenol levels, i.e., between the BSG L and the BSG D as expected. Since BSG Mix constituted both the L and D (~9:1 *w*/*w*) BSG, we also measured the polyphenols in its crude and various ‘liquor’ fractions (prior to neutralisation and EtOAc partitioning) by UPLC-MS/MS. The crude extract of the BSG Mix contained low levels of polyphenols (~26 µg/g BSG dw), of which catechin constituted more than 50% of the total free polyphenols. This was 45- to 54- fold less than the total polyphenols present in the various EtOAc fractions (1170–1387 µg/g dw) of the same sample. McCarthy et al. [25] have also reported low levels of total polyphenols (30.6 µg/g in light and 27.2 µg/g in dark BSG dw) using HPLC coupled with diode array detector (DAD)-mass spectrometry analysis of the crude extracts. Stefanello et al. [15], on the other hand, have recorded 82.4 µg/g total polyphenols in the crude BSG extract, of which catechin constituted 83% of the total polyphenols. The TPC for these two studies ranged from 0.98–4.53 mg GAE/g BSG dw, which corroborate our findings. An even more interesting finding is that the total polyphenols in the liquors of BSG Mix were significantly lower than in the corresponding EtOAc fractions despite the fact that the TPC values for all ‘liquor’ fractions were very high (Table 1 and Table 3). A similar observation was made by Stefanello et al. [15], where the TPC for the liquor was 17.4 mg GAE/g BSG dw, whilst the HPLC-DAD quantification of total polyphenols for the same liquor was 3195 µg/g dw. The HPLC-DAD value was closer to the TPC value of their crude BSG extract (3.43 mg GAE/g BSG dw). The high TPC values in the liquor fractions must have been attributed by other non-polyphenolic compounds such as reducing sugars, amino acids and peptides [4] that get fractionated in the water part during the EtOAc:water partitioning.

In all the saponified BSG extracts, ferulic acid was the most predominant phenolic acid comprising in excess of 50% of the total polyphenols followed by *p*-coumaric acid. When the most abundant phenolic acid, i.e., ferulic acid is considered, there is no significant difference between the efficiency of the different extraction methodologies within the same type of BSG substrate. Several other studies have also established that the dominant polyphenols in BSG are ferulic acid and *p*-coumaric acid [10,26] and thus had become the target compounds of recovery in several studies [9,10,11,22,39,40,41,42]. Other abundant polyphenols in the BSG were sinapic acid and syringic acid, which have also been reported by other authors [25,42].

The UPLC-MS/MS determination of total polyphenols from MAE and UAE of the BSG EtOAc fractions showed a similar trend to their TPC values (Table 1 and Table 3), where MAE and UAE yielded lower total polyphenols than the conventional maceration method. The lowest recovery of total polyphenols was by the MAE method. As explained earlier in Section 3.1, the MAE technique was not able to overcome the lignin-rich barrier, and that the extraction parameters used in the MAE and UAE may have induced thermal degradation of polyphenols.

The UPLC-MS/MS quantification of polyphenols in the various BSG EtOAc fractions was closer to the spectrophotometric FC-method (Table 3 vs. Table 1). Athanasios et al. [43] have used gas chromatography-mass spectrometry (GC-MS) and showed total polyphenols ranged between 2688 to 4884 µg/g dw in the four different batches of BSG, although the authors did not perform spectrophotometric analysis but these values are very close to TPC values of BSG in general.

## 4. Conclusions

The UPLC-MS/MS data have shown that the saponification followed by acidification and subsequent liquid-liquid partitioning (EtOAc) is the best procedure for polyphenol recovery and enrichment from BSG irrespective of extraction method. Without neutralisation and partitioning, the colourimetic chemical method falsely overestimates the total phenolic content and levels quantified by related assays in the liquors. Hyphenated chromatographic quantification methods such as LC-MS/MS is therefore necessary to accurately portray levels of total BSG polyphenols present. UAE and MAE treatments did not improve the BSG polyphenol yield indicating the thermal degradation of polyphenols with the extraction parameters used in these systems. The findings also suggest that ultrasonic bath operating at 35 kHz is less efficient in aqueous solution for the extraction of polyphenols from BSG. However, these techniques may improve the polyphenol yield and efficacy with further optimisation and when used with other systems, such as ultrasonic probes, and in combination with appropriate organic solvents.

## Figures and Tables

**Table 1:** Total phenolic contents in mg GAE/g BSG dw in the NaOH saponified BSG extracts (liquors) and their subsequent ethyl acetate fractions following neutralisation (EtOAc); Ctrl represents maceration method, microwave assisted extraction (MAE), ultrasound assisted extraction (UAE) of light (L), dark (D) and Mix BSG. For each substrate, total phenolic content (TPC) values bearing different letters (a, b, c) are significantly different (*p* < 0.05) from each other. Shadow is to make the data distinguishable between the samples.

Samples	TPC mgGAE/g BSG dw		
	BSG L	BSG D	BSG Mix
Crude	2.84 ± 0.11 ^c^	2.81 ± 0.26 ^c^	3.85 ± 0.04 ^c^
Liquor Ctrl	16.67 ± 0.87 ^b^	17.27 ± 0.41 ^ab^	19.20 ± 0.40 ^a^
Liquor Ctrl EtOAc	4.67 ± 0.27 ^c^	3.08 ± 0.15 ^c^	4.71 ± 0.28 ^c^
Liquor MAE	15.42 ± 1.16 ^b^	15.55 ± 0.56 ^b^	16.94 ± 1.84 ^b^
Liquor MAE EtOAc	3.85 ± 0.19 ^c^	3.01 ± 0.19 ^c^	4.24 ± 0.22 ^c^
Liquor UAE	15.76 ± 0.72 ^b^	16.72 ± 0.96 ^b^	16.99 ± 0.32 ^b^
Liquor UAE EtOAc	4.17 ± 0.21 ^c^	3.43 ± 0.46 ^c^	4.62 ± 0.27 ^c^

**Table 2:** HPLC-Q-ToF identification of polyphenols in the ethyl acetate fraction of hydrolysed light BSG.

Peak No.	RT (min.)	Observed [M − H]^−^ (*m*/*z*)	Calculated [M − H]^−^ (*m*/*z*)	Chemical Formula	MS/MS Fragment Ions (*m*/*z*)	Tentative Identification
1	2.05	153.0169	153.0188	C_7_H_6_O_4_	109.03	protocatechuic acid
2	3.50	137.0227	137.0239	C_7_H_6_O_3_	93.04	hydroxybenzoic acid
3	4.93	179.0331	179.0344	C_9_H_8_O_4_	135.04	caffeic acid
4	5.43	197.0452	197.0450	C_9_H_10_O_5_	153.03	syringic acid
5	5.65	121.0282	121.0290	C_7_H_5_O_2_	92.03	benzoic acid
6	6.80	163.0380	163.0395	C_9_H_8_O_3_	119.05	coumaric acid
7	7.13	387.1073	387.1080	C_20_H_20_O_8_	343.13, 193.05, 178.03, 149.07, 134.05	ferulic-ferulic acid dimer
8	7.34	223.0614	223.0606	C_27_H_30_O_16_	179.02	sinapic acid
9	7.54	341.1019	341.1025	C_19_H_18_O_6_	267.08, 193.05, 134.04	decarboxylated diferulic acid
10	7.87	385.0915	385.0923	C_20_H_18_O_8_	282.09, 267.07 (100%), 239.08, 148.06	diferulic acid
11	8.73	385.0909	385.0923	C_20_H_18_O_8_	325.09/326.09, 282.11/281.11 (100%), 267.08 (75%).	diferulic acid isomer
12	9.19	193.0516	193.0501	C_10_H_10_O_4_	178.03, 134.04	ferulic acid
13	9.39	577.1342	577.1346	C_30_H_26_O_12_	533.17, 355.09,	triferulic acid
14	10.44	341.1035	341.1025	C_19_H_18_O_6_	326.09, 311.07, 282.09, 267.08 (100%), 239.08	decarboxylated diferulic acid isomer

**Table 3:** UPLC-TQD quantification of BSG polyphenols *.

Samples	Ferulic Acid	*p*-Coumaric Acid	Catechin	4-Hydroxybenzoic Acid	Sinapic Acid	Syringic Acid	Protocatechuic Acid	Caffeic Acid	Total
**BSG L Ctrl EtOAc**	1809.5 ± 272.8 ^a^	686.6 ± 59.0 ^a^	2.11 ± 0.23 ^b^	16.66 ± 4.45 ^a^	14.63 ± 2.48 ^a^	33.9 ± 10.44 ^b^	3.46 ± 1.04 ^ab^	0.147 ± 0.065 ^d^	2741.1 ± 5.2 ^a^
**BSG L MAE EtOAc**	1545.6 ± 157.3 ^a^	499.1 ± 31.2 ^bc^	1.43 ± 0.48 ^b^	9.41 ± 1.15 ^bcd^	11.02 ± 3.99 ^ab^	18.9 ± 7.26 ^bc^	1.38 ± 0.72 ^cd^	0.370 ± 0.031 ^b^	2087.2 ± 196.8 ^a^
**BSG L UAE EtOAc**	1669.7 ± 21.8 ^a^	579.2 ± 22.7 ^b^	1.05 ± 0.07 ^b^	10.76 ± 0.99 ^bcd^	10.36 ± 1.52 ^ab^	17.8 ± 3.68 ^bc^	2.29 ± 0.83 ^bc^	0.176 ± 0.013 ^d^	2291.2 ± 42.7 ^ab^
**BSG D Ctrl EtOAc**	404.7 ± 51.0 ^cd^	185.3 ± 8.3 ^f^	1.66 ± 1.01 ^b^	13.12 ± 0.38 ^ab^	7.63 ± 1.92 ^bc^	76.4 ± 28.84 ^a^	3.83 ± 0.63 ^a^	0.407 ± 0.065 ^b^	693.0 ± 85.7 ^de^
**BSG D MAE EtOAc**	351.0 ± 33.9 ^d^	155.3 ± 7.5 ^f^	1.23 ± 0.33 ^b^	11.36 ± 2.28 ^bc^	4.68 ± 0.67 ^c^	21.7 ± 4.84 ^bc^	4.09 ± 0.55 ^a^	0.547 ± 0.079 ^a^	549.9 ± 41.5 ^e^
**BSG D UAE EtOAc**	413.6 ± 135.8 ^cd^	173.4 ± 56.6 ^f^	2.18 ± 0.74 ^b^	10.69 ± 1.39 ^bcd^	8.28 ± 0.46 ^bc^	17.3 ± 5.91 ^bc^	4.85 ± 0.47 ^a^	0.389 ± 0.052 ^b^	629.9 ± 190.9 ^de^
**BSG Mix Ctrl EtOAc**	894.6 ± 82.8 ^b^	476.4 ± 35.1 ^bcd^	nd	6.02 ± 0.93 ^de^	9.59 ± 0.23 ^abc^	nd	0.062 ± 0.012 ^d^	0.226 ± 0.049 ^cd^	1387.0 ± 119.0 ^c^
**BSG Mix MAE EtOAc**	796.8 ± 68.1 ^b^	355.4 ± 33.0 ^e^	0.47 ± 0.82 ^b^	6.88 ± 0.30 ^cde^	10.23 ± 0.68 ^ab^	nd	0.015 ± 0.026 ^d^	nd	1169.8 ± 66.4 ^c^
**BSG Mix UAE EtOAc**	848.5 ± 15.2 ^b^	386.9 ± 6.7 ^de^	nd	6.59 ± 0.55 ^de^	11.33 ± 1.54 ^ab^	nd	0.174 ± 0.085 ^d^	0.328 ± 0.005 ^bc^	1253.8 ± 11.3 ^c^
**BSG Mix Crude**	2.8 ± 2.41 ^e^	nd	14.05 ± 1.19 ^a^	0.11 ± 0.12 ^f^	8.28 ± 0.14 ^bc^	nd	0.49 ± 0.17 ^d^	nd	25.7 ± 1.97 ^f^
**BSG Mix Liquor Ctrl**	714.1 ± 76.7 ^bc^	423.3 ± 17.6 ^cde^	1.09 ± 0.98 ^b^	4.24 ± 0.50 ^ef^	12.29 ± 1.09 ^ab^	nd	nd	nd	1155.0 ± 93.2 ^c^
**BSG Mix Liquor MAE**	647.4 ± 40.7 ^bcd^	330.6 ± 49.5 ^e^	1.86 ± 0.36 ^b^	4.26 ± 0.33 ^ef^	9.52 ± 0.29 ^bc^	nd	nd	nd	993.6 ± 74.8 ^cd^
**BSG Mix Liquor UAE**	739.1 ± 22.3 ^b^	371.9 ± 30.9 ^de^	nd	4.12 ± 0.37 ^ef^	11.11 ± 0.^39 ab^	nd	nd	nd	1126.3 ± 53.2 ^c^

* Values are expressed as µg/g BSG dw (mean ± SD); nd—not detected; For each substrate, the values reported, for each individual and total polyphenols in crude, liquors and their ethyl acetate (EtOAc) fractions bearing different letters (a, b, c, d, e, f) are significantly different (*p* < 0.05) from each other. Shadow is to make the data distinguishable between the samples.

## Supplementary Materials

The following is available online at https://www.mdpi.com/2076-3921/8/9/380/s1, Table S1: Multiple reaction monitoring (MRM) transitions, cone voltages and collision energies used for the UPLC-TQD quantification of BSG polyphenols. Figure S1: Extraction ion chromatograms for peak 1 (*m*/*z* 153.017 [M − H]^−^), peak 3 (*m*/*z* 179.0133 [M − H]^−^) and peak 4 (*m*/*z* 197.045 [M − H]^−^).
