# Supplementary material for: Recovery of Polyphenols from Brewer’s Spent Grains
Source: Antioxidants (Basel). 2019 Sep 7;8(9):380. doi: 10.3390/antiox8090380 (PMC6769810; doi:10.3390/antiox8090380)
Supplement: Supplementary file 1 [file antioxidants-08-00380-s001.pdf]

antioxidants

MDPI

Article

Recovery of Polyphenols from Brewer's Spent Grains

Rares I. Birsan[1,2], Peter Wilde[2], Keith W. Waldron[3] and Dilip K. Rai [1,*]

[1] Department of Food BioSciences, Teagasc Food Research Centre Ashtown, Dublin, D15KN3K, Ireland;
[2] Food Innovation and Health Programme, Quadram Institute Bioscience, Norwich Research Park, NR4 7UQ, United Kingdom;
[3] Anglia Science Writing Ltd., Wramplingham, Norfolk, NR18 0RU, United Kingdom
* Correspondence: dilip.rai@teagasc.ie; Tel.: +353-(0)1-805-9500

Table S1. Multiple reaction monitoring (MRM) transitions, cone voltages and collision energies used for the UPLC-TQD quantification of BSG polyphenols.

Polyphenol	MRM transitions (*m/z*)	Cone Voltage (V)	Collision energy (eV)
Ferulic acid	192.9→133.9<br>192.9→177.9	31	16<br>12
p-coumaric acid	163.0→118.9<br>163.0 → 92.9	25	14<br>30
Catechin	289.1→ 245.0<br>289.1→ 108.9	40	16<br>22
Syringic acid	197.1→152.9<br>197.1→181.9	31	12<br>14
4-hydroxybenzoic acid	137.0→64.9<br>137.0→92.9	29	26<br>14
Sinapic acid	223.1→120.9<br>223.1→163.9	32	26<br>14
Caffeic acid	179.0→78.9<br>179.0→134.9	35	24<br>16
Protocatechuic acid	153.0→80.9<br>153.0→108.9	29	8<br>14

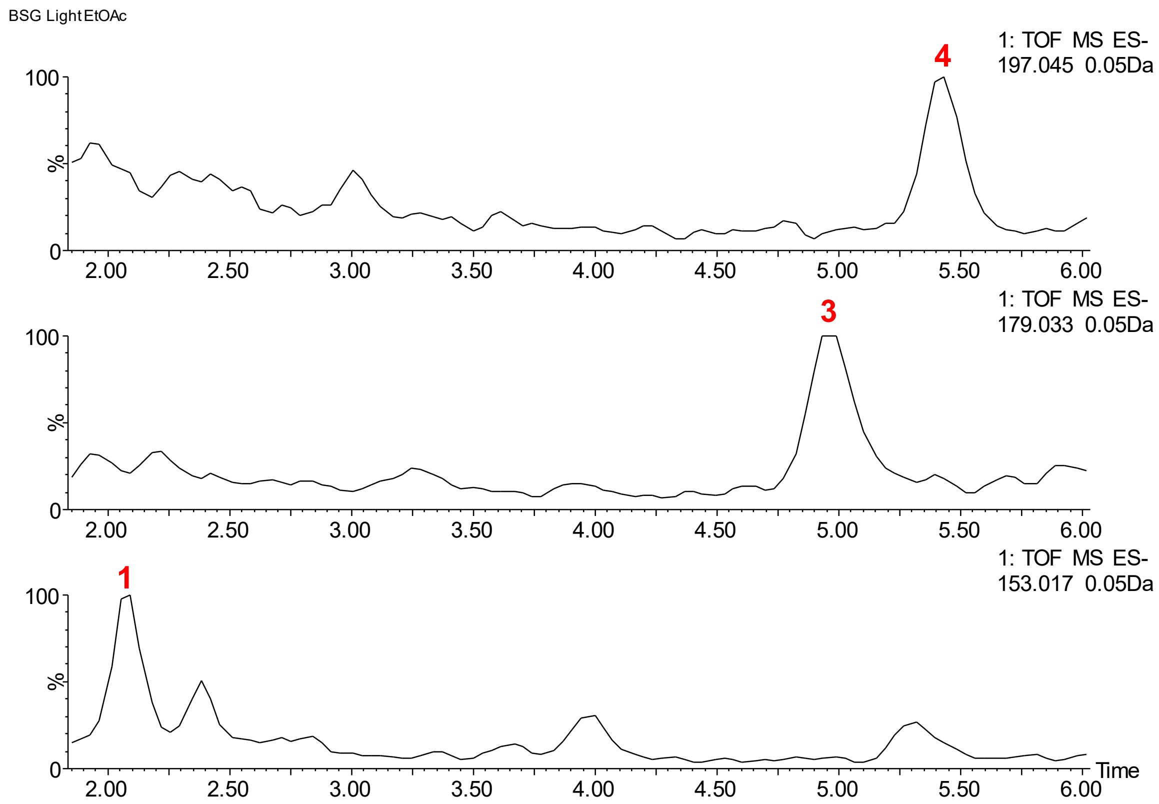

Figure S1. Extraction ion chromatograms for peak 1 (*m*/*z* 153.017 [M-H]$^-$), peak 3 (*m*/*z* 179.0133 [M-H]$^-$) and peak 4 (*m*/*z* 197.045 [M-H]$^-$).
